# Bioaerosol biomonitoring: Sampling optimization for molecular microbial ecology

**DOI:** 10.1111/1755-0998.13002

**Published:** 2019-04-20

**Authors:** Robert M. W. Ferguson, Sonia Garcia‐Alcega, Frederic Coulon, Alex J. Dumbrell, Corinne Whitby, Ian Colbeck

**Affiliations:** ^1^ School of Biological Sciences University of Essex Colchester UK; ^2^ School of Water, Energy and Environment Cranfield University Cranfield UK

**Keywords:** airborne microorganisms, bioaerosol sampling, biomonitoring, filters, impingement, next ‐ sequencing

## Abstract

Bioaerosols (or biogenic aerosols) have largely been overlooked by molecular ecologists. However, this is rapidly changing as bioaerosols play key roles in public health, environmental chemistry and the dispersal ecology of microbes. Due to the low environmental concentrations of bioaerosols, collecting sufficient biomass for molecular methods is challenging. Currently, no standardized methods for bioaerosol collection for molecular ecology research exist. Each study requires a process of optimization, which greatly slows the advance of bioaerosol science. Here, we evaluated air filtration and liquid impingement for bioaerosol sampling across a range of environmental conditions. We also investigated the effect of sampling matrices, sample concentration strategies and sampling duration on DNA yield. Air filtration using polycarbonate filters gave the highest recovery, but due to the faster sampling rates possible with impingement, we recommend this method for fine ‐scale temporal/spatial ecological studies. To prevent bias for the recovery of Gram‐positive bacteria, we found that the matrix for impingement should be phosphate‐buffered saline. The optimal method for bioaerosol concentration from the liquid matrix was centrifugation. However, we also present a method using syringe filters for rapid in‐field recovery of bioaerosols from impingement samples, without compromising microbial diversity for high ‐throughput sequencing approaches. Finally, we provide a resource that enables molecular ecologists to select the most appropriate sampling strategy for their specific research question.

## 1 INTRODUCTION

Bioaerosols are biogenic aerosols (size range: ~0.05–100 µm), comprising material released by organisms (e.g., spores, pollen, volatile organic metabolites and endotoxins), live and dead microorganisms, and cell fragments. One of the main drivers for the study of bioaerosols is their potential threat to the environment and human health. Once inhaled, bioaerosols are associated with a wide range of negative health effects (e.g., infectious disease, allergies, asthma, cancer and acute toxicity), and are a key concern in the biowaste and recycling industry (Bush & Portnoy, 2001; Douwes, Thorne, Pearce, & Heederik, 2003; Gladding & Gwyther, 2017; Gladding, Thorn, & Stott, 2003; Kim, Kabir, & Jahan, 2018; Pankhurst et al., 2009; Pearson et al., 2015; Wéry, 2014). In addition to public health issues, bioaerosols play significant roles in atmospheric chemistry and meteorology, and contain dispersing microbes and propagules from higher taxa, underpinning metacommunity dynamics and species distributions (Ariya & Amyot, 2004; Ariya et al., 2009; Bauer et al., 2003; Estillore, Trueblood, & Grassian, 2016; Fröhlich‐Nowoisky et al., 2016; Iannone, Chernoff, Pringle, Martin, & Bertram, 2011). While a great deal of research has focused on fungal pathogens (e.g., *Aspergillus fumigatus*: Douglas et al., 2017; Recer, Browne, Horn, Hill, & Boehler, 2001; Williams, Douglas, Roca Barcerlo, Hansell, & Hayes, 2019), bacterial bioaerosols represent an urgent research priority due to their role in disease outbreaks (Van Leuken et al., 2016; Weiss, Boyd et al., 2017; Weiss, Xu et al., 2017).

Despite bioaerosols being an important transmission route for infectious and sensitization agents, information on the microbiological components of bioaerosols from different environments is scarce (Blais‐Lecours, Perrott, & Duchaine, 2015). This lack of knowledge hampers our ability to address both key public health (Douglas, Hayes et al., 2017; Douglas, Tyrrel et al., 2017; Pearson et al., 2015; WHO, 2009) and broad ecological questions, relating to species dispersal and biogeography (Clark et al., 2017; Dumbrell, Nelson, Helgason, Dytham, & Fitter, 2010a, 2010b ; Zhou & Ning, 2017). Traditionally, bioaerosols have been studied using culture‐based methods, but culturing captures only a small fraction of the total microbial community. In aquatic and terrestrial environments, molecular methods are routinely used (Clark et al., 2018; Mommer, Dumbrell, Wagemaker, & Ouborg, 2011) and could also provide a rapid, sensitive and specific approach to analysing airborne microorganisms, especially from low‐concentration environments (Colbeck & Whitby, [Link] press). Yet, optimum methods for collecting nucleic acid material from air samples remains under investigation, and standardized sampling procedures have not yet been established (Hoisington, Maestre, King, Siegel, & Kinney, 2014; Mbareche, Brisebois, Veillette, & Duchaine, 2017; Reponen, 2017).

Due to the low environmental concentrations of bioaerosols, collecting sufficient genetic material for molecular methods is problematic and is dependent on the biomass present, which varies between environments. Consequently, collection devices with high flow rates or high collection efficiencies are necessary (Morgan, Darling, & Eisen, 2010). Moreover, nucleic acid yield, and hence microbial diversity recovered, will depend on the nucleic acid extraction protocol used (Luhung et al., 2015; Morgan et al., 2010; Peccia & Hernandez, 2006). This is particularly important for low‐biomass bioaerosol samples, where the DNA extraction method applied needs to have high recovery efficiencies (Morgan et al., 2010). Indeed, often only dominant sequences are recovered and the “rare biosphere” is missed (Colbeck & Whitby, [Link] press). Characterizing microorganisms from bioaerosols is further hindered by high concentrations of PCR inhibitors (e.g., humic acids and inorganic particles), which impede downstream molecular analysis (Luhung et al., 2015; Peccia & Hernandez, 2006). The matrix used for collection is also known to introduce biases (Adams, Tian et al., 2015; Aguayo, Fourrier‐Jeandel, Husson, & Ioos, 2018; Castaño et al., 2017; Wang et al., 2015). Thus, it is difficult to compare bioaerosol studies, due to differences in samplers, collection time, airflow rate and analysis methods, and consequently many basic questions remain unanswered. For example, how does sampler choice influence the results? How long/what volume of air shoud be sampled? How does filter/liquid matrix affect DNA yield? How should the sample be concentrated for analysis?

The three most common methods for bioaerosol sampling are air filtration, liquid impingement and impaction, all of which have been successfully used for collecting bioaerosol environmental DNA (Galès et al., 2015; Mayol, Jiménez, Herndl, Duarte, & Arrieta, 2014; Pankhurst et al., 2012). Filters typically have high collection efficiencies (>95%) for particles >0.5 µm in diameter and are simple to use (Lee & Mukund, 2001; Miaskiewicz‐Peska & Lebkowska, 2012). There are three main classes of filter: fibrous, membrane and flat filters. In this study, we used a representative of the most commonly used filter classes: glass fibre (GF), polycarbonate (PC) and gelatin (Gel). Fibrous filters trap particles within a matrix of randomly orientated fibres (e.g., glass fibre and cellulose). Membrane filters have a complex internal structure of pores within which particles are deposited (e.g., gelatin and polyvinyl chloride). Flat filters collect particles on the filter surface with the air passing through pores in the membrane (e.g., polycarbonate). The way the filter traps the particle influences both what is collected (e.g., spores or cells) and how easily it is released for downstream analysis (Burton, Adhikari, Grinshpun, Hornung, & Reponen, 2005; Duquenne, Coulais, Bau, & Simon, 2018; Dybwad, Skogan, & Blatny, 2014a; Yoo et al., 2017). The advantages of filters for molecular analyses are that the captured microorganisms remain viable and nucleic acid extraction occurs directly from the filter (Yoo et al., 2017). However, one problem with using filters for molecular methods is that spore‐forming microorganisms may be preferentially recovered, depending on filtration time, pore size and filter type (Yoo et al., 2017).

Impactors collect particles by depositing them onto a surface transverse to the airflow. The main advantage is that the particle size collected can be controlled by varying the flow rate (which is typically between 10 and 700 L/min). However, with impactors, cell viability is lost due to impact stress and recovery efficiency is often reduced due to low flow rates and particle bounce (Griffin, 2007). Impingers use a cyclone to deposit bioaerosols into a liquid. Impingers generally have lower collection efficiencies than filters, especially for small particles (~50% at 0.5–5 µm and ~90% at 10 µm; Carvalho et al., 2008; Dybwad et al., 2014a) but airflow rates for impingers are generally higher (300–600 L/min, compared to 2–300 L/min for filtration), permitting shorter sampling periods. Impingement also overcomes the problems associated with organism desiccation that can occur with filters and impactors. However, depending on the liquid matrix used, cell growth and lysis during storage have a demonstrable effect on culture‐based studies (Chang & Wang, 2015), but it is unknown if this is true for molecular studies, nor what the optimum method for concentrating samples for downstream nucleic acid extraction after impingement is.

**Figure 1:**
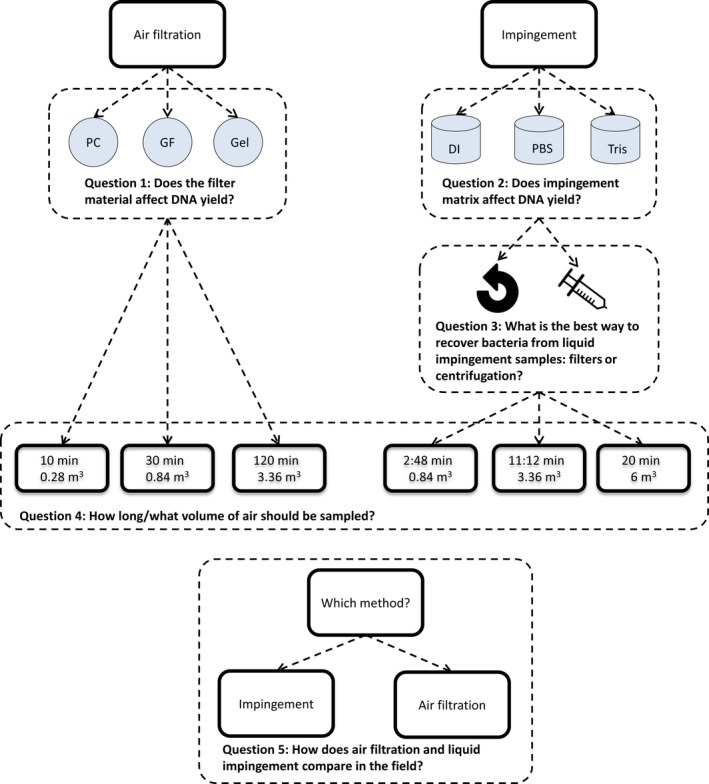
Graphical summary of the research questions addressed for aerosol biomonitoring. DI: deionized water; GF: glass fibre filter; Gel: gelatine filter; PBS: phosphate ‐buffered saline; PC: polycarbonate filter; Tris: Tris (HCl) buffer [Colour figure can be viewed at http://wileyonlinelibrary.com]

Here, we investigated the optimal methods for the biomonitoring of bacterial bioaerosol samples. Specifically, we evaluated the suitability of air filtration and liquid impingement as bioaerosol collection methods across differing environmental settings and temporal/spatial scales. We compared GF, PC and Gel filters in relation to DNA yield. We also investigated the effect of three liquid matrices for use with liquid impingement, namely deionized water (DI), phosphate‐buffered saline (PBS) and Tris hydrochloride buffer (Tris‐HCl), and whether biomass recovery was greater with centrifugation or filtration. Finally, we provide a new resource that enables molecular ecologists and air regulators to select the most appropriate bioaerosol sampling strategy for their research questions. We address the following questions (summarized in Figure 1):

1. Does filter type affect DNA yield?
2. Does the liquid impingement matrix affect DNA yield?
3. What is the best way to recover bacteria from liquid impingement samples—filtering or centrifugation?
4. For how long and what volume of air should be sampled to obtain sufficient DNA yields for downstream molecular processing in different environmental contexts?
5. How do DNA yields vary between air filtration and liquid impingement sampling methods when applied in different environments?

## 2 MATERIALS AND METHODS

**Figure 2:**
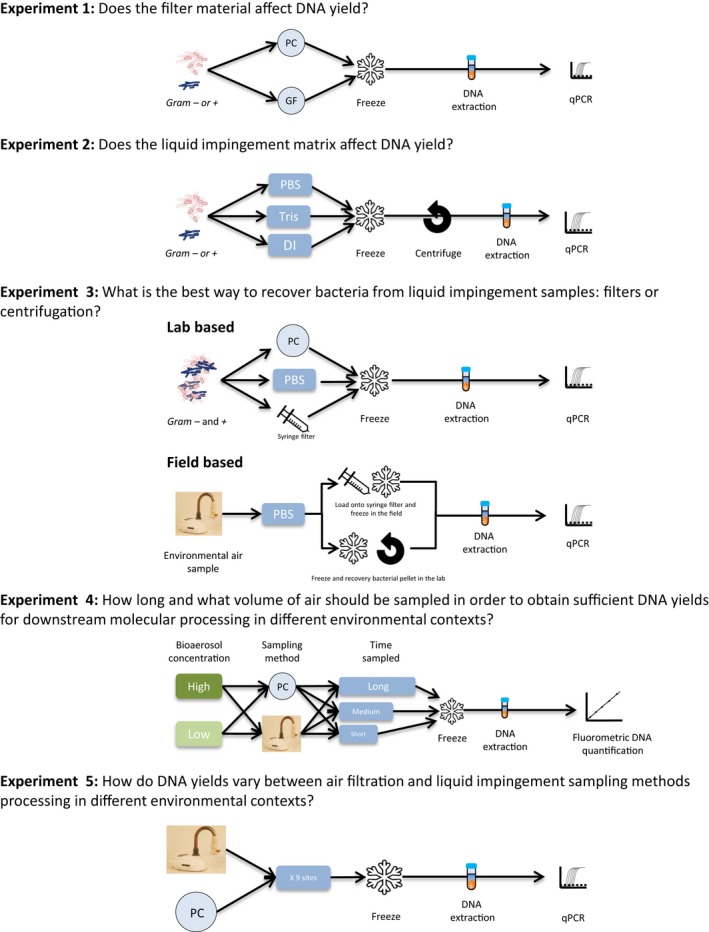
Overview of the five experiments carried out in this study. Gel filters were excluded from Experiment 1 due to DNA contamination (See Results section)

Five experiments were designed (Figure 2) to address the questions outlined in the introduction (Figure 1). All conditions of Experiments 1, 2 and 3 (Lab Experiment) were carried out twice, in triplicate (*n* = 3) to determine reproducibility. For all experiments, we evaluated performance primarily based on the maximum DNA recovery, using qPCR of the 16S rRNA gene for Experiments 1–3 and 5. For Experiment 4 we used direct measurement of total DNA with a fluorospectrometer as knowing the numbers of 16S rRNA copies would not have be relevant to metagenomic workflows. For Experiment 3 we also compared bacterial diversity between the methods with high‐throughput amplicon sequencing of the 16S rRNA gene.

### 2.1 Experimental procedures

#### 2.1.1 Experiment 1 addresses Question 1: Does the filter material affect DNA yield?

Three filters were tested: PC (Cyclopore, Whatman, Fisher), GF (FisherBrand, Fisher), and Gel pre‐sterilized by gamma irradiation (Sartorius). All filters were 47 mm in diameter with a 0.4‐µm pore size. GF and PC filters were wrapped in foil and sterilized by autoclaving (121°C for 20 min). Filters were placed in sterile Petri dishes (Fisher) and either *Escherichia coli* DH5α (representative Gram negative) or *Bacillus subtilis* (representative Gram positive; obtained from Essex Culture Collection) at 1 × 10^7^ colony‐forming units (CFU)/ml was added in ten 1‐µl aliquots evenly across the surface of each filter (see Supporting information Appendix S1 for bacterial growth conditions). Filters were then rolled (so the bacteria were on the inside surface), placed into 2‐ml microcentrifuge tubes and stored at −20°C overnight to simulate storage after sampling. Before DNA extraction, all filters were thawed to room temperature. To prevent the filters from obstructing bead lysis, the following was performed: (a) the PC filters were placed into the lysis tubes intact; (b) the GF filters were placed into a Petri dish, cut into fifths and placed into lysis tubes; and (c) the Gel filters were fragmented using sterile tweezers and then placed in the lysis tubes. Procedural blanks comprising empty tubes and sterile filters were also included. To determine the DNA extraction efficiency, 10 µl of each bacterial culture (at 1 × 10^7^ CFU/ml) was added directly to a lysis tube. DNA extraction and qPCR followed methods described in the Supporting information Appendix S1 and qPCR analysis section (below) respectively. All conditions were carried out twice, in triplicate (*n* = 3) to determine reproducibility.

#### 2.1.2 Experiment 2 addresses Question 2: Does the liquid impingement matrix affect DNA yield?

Three buffers were prepared as follows: PBS (10 mM PO_4_^3−^, 137 mM NaCl and 2.7 mM KCl, pH 7.4); Tris‐HCl (1 M Tris base) adjusted to pH 8 with HCl; and DI water. All buffers were sterilized by autoclaving (121°C for 20 min). Then, 10 µl of 1 × 10^7^CFU/ml of either *E. coli* (Gram negative) or *B. subtilis* (Gram positive) were aseptically pipetted into 10 ml of each buffer and the mixtures were stored at −20°C overnight to simulate storage after sampling. The culture–buffer mixtures were defrosted at room temperature and centrifuged at 3,395 *g* for 45 min, pellets were re‐suspended in 500 µl 1% (v/v) sodium dodecylsulphate buffer by aspiration in a 1‐ml pipette, vortexed for 2 × 10 s and transferred to a bead‐lysis tube. For each culture, 10 µl (at 1 × 10^7^CFU/ml) was also added directly to a lysis tube to determine the DNA extraction efficiency. Procedural controls (comprising buffer with no culture) were also performed. DNA extraction and qPCR followed methods described in the Supporting information Appendix S1 and qPCR analysis section (below) respectively. All conditions were carried out twice, in triplicate (*n* = 3) to determine reproducibility.

#### 2.1.3 Experiment 3 applied lab and field experiments (Figure 2) to address Question 3: What is the best way to recover bacteria from liquid impingement samples: filtering or centrifugation?

In the lab experiment, based on the data obtained from Experiments 1 and 2, the methods determined to be optimal (i.e. PC filters, and pelleting in PBS) were compared to a rapid in‐field method using a syringe filter. In all cases, 10 µl of a mixed culture of *E. coli* (Gram negative) and *B. subtilis* (Gram negative) (at 1 × 10^11^CFU/ml) was added to either PC filters or PBS and stored overnight at − 20°C. For syringe filters, 10 µl of the bacterial mixture (at 1 × 10^11^CFU/ml) was added to 10 ml of PBS, and filtered through a pre‐sterilized syringe filter (Minisart, 0.22 µm, Sartorius) that was then sealed at both ends with foil and stored overnight at −20°C to simulate post‐sampling storage. DNA was extracted from the PC filters and PBS as described previously. To extract DNA from the syringe filters, they were cut with electrical wire cutters (sterilized in 1% [w/v] sodium hypochlorite) and the filters were removed and placed into a lysis tube. Procedural controls (comprising buffer with no culture) were also performed. DNA extraction and qPCR followed methods described in the Supporting information Appendix S1 and qPCR analysis section (below) respectively. All conditions were carried out twice, in triplicate (*n* = 3), to determine reproducibility.

In the field experiment, air samples were collected from two field sites: University of Essex (UOE) (51°52′36.912″N, 0°56′34.6308″E) and a wheat farm near Birch, Essex, UK (51°47′32.2908″N, 0°50′38.2632″E). Six replicate samples were collected at each location and alternate samples were processed by either centrifugation or syringe filtering. Air samples were collected with a Coriolis µ wet cyclone impinger (Bertin, Air Monitors) into 15 ml PBS at 300 L/min for 10 min. The sampling cones were sterilized in 10% (w/v) sodium hypochlorite for 24 hr followed by a second wash in Milton sterilizing liquid (Rivadis) diluted at 1:80 in sterile DI water (active ingredients: 1% [w/v] sodium hypochlorite and 16.5% [w/v] NaCl). Preliminary optimization showed that this is sufficient to remove residual DNA from cones after spiking with pure cultures (Supporting information Figure S1). The syringe filter samples were immediately filtered. All samples were immediately frozen on dry ice and stored at −20°C for ≤2 weeks before DNA extraction. DNA extraction and qPCR followed methods described in the Supporting information Appendix S1 and qPCR analysis section (below) respectively. HiSeq sequencing was performed as described in the DNA sequencing section below. Bioinformatics analysis was performed in qiime (Caporaso et al., 2010) and cited standalone packages: sickle (Joshi & Fass, 2011), spades (Bankevich et al., 2012), bayeshammer (Nikolenko, Korobeynikov, & Alekseyev, 2013), pear (Zhang, Kobert, Flouri, & Stamatakis, 2014), pandaseq (Masella, Bartram, Truszkowski, Brown, & Neufeld, 2012), vsearch (Rognes, Flouri, Nichols, Quince, & Mahé, 2016), uchime (Edgar, Haas, Clemente, Quince, & Knight, 2011), rdp classifier (Wang, Garrity, Tiedje, & Cole, 2007), as described by Dumbrell, Ferguson, and Clark (2017), Ferguson, Gontikaki, Anderson, and Witte (2017) and detailed in the Supporting information Appendix S1.

#### 2.1.4 Experiment 4 addresses Question 4: How long and what volume of air should be sampled in order to obtain sufficient DNA yields for downstream molecular processing in different environmental contexts?

Air samples were collected in triplicate (*n* = 3) from two field sites of differing bioaerosol concentrations: a green waste open windrow compost site near Birch, UK (51°50'33.9972''N, 0°46'27.2784''E, high bioaerosol concentration), and an urban garden in Colchester UK, (51°52′53.9184″N, 0°53′9.0204″E, low bioaerosol concentration). Air samples were either collected with a Coriolis µ wet cyclone impinger (Bertin, Air Monitors) or onto PC filters attached to a vacuum pump (Cole‐Palmer) as previously described. Three different sampling times/volume of air were used for each sampling method in parallel (Table 1) at each location in triplicate. The filters were fully submerged in 0.5 ml RNAlater (Qiagen), placed immediately on dry ice and stored for ≤2 weeks at −80°C prior to DNA extraction. DNA was extracted using a DNeasy PowerSoil Kit (Qiagen) according to the manufacturer's instructions. DNA quantification was performed using the Quant‐iT dsDNA assay kit (Thermo Fisher Scientific) with a FLUO star Omega fluorospectrometer plate reader (BMG Labtech) and a five‐point triplicate standard curve, with an *R* ^2^ > 0.99. Thresholds for DNA yield required for metagenomic sequencing were set based on current guidelines for preparation of libraries for metagenome sequencing with the Nextera DNA Library Prep Reference Guide (Illumina), which are 50 ng of DNA per sample, or using the Nextera XT DNA Library Prep kit which requires 1 ng of DNA per sample.

**Table 1:** Summary of sampling periods and volume of air collected

Time (min:s)		Volume of air (m^3^)	
Filtration	Impingement	Filtration	Impingement
10:00		0.28	
30:00	02:48	0.84	0.84
120:00	11:12	3.36	3.36
	20:00		6

#### 2.1.5 Experiment 5 addresses Question 5: How do DNA yields vary between air filtration and liquid impingement sampling methods processing in different environmental contexts?

Air samples were collected at nine sites (A–I) in southeast England, comprising a mixture of urban, industrial and agricultural locations (Supporting information Table S1). At each site, nine sets of triplicate 20‐min samples were collected by liquid impingement using a Coriolis µ wet cyclone impinger (Bertin, Air Monitors) as described previously. At each site, three sets of triplicate air samples were collected by air filtration onto PC filters at 28 L/min for 120 min using a Gast vacuum pump (Cole‐Palmer) with the filters placed in 47‐mm Swin‐Lok plastic filter holders (Whatman). The sampling cones and filter holders were sterilized in 1% (w/v) sodium hypochlorite and Milton liquid as described previously. All samples were frozen on dry ice and stored at − 20°C. DNA extraction and qPCR followed methods described in the Supporting information Appendix S1 and qPCR analysis section (below) respectively. The concentration of 16S rRNA gene copies was normalized for the volume of air sampled and compared across concurrent samples at the same site to determine differences in yield between methods.

### 2.2 qPCR analysis of the 16S rRNA genes

DNA standards for qPCR analysis were created from PCR‐amplified *E. coli*, *B. subtilis*, a mixture of the two, or an environmental DNA extract to match samples being quantified using the general bacterial 16S rRNA gene V3–V4 primer pair S‐D‐Bact‐0341 and reverse Primer S‐D‐Bact‐0785‐a‐A‐21 (Klindworth et al., 2013). PCR mixtures for preparing DNA standards (total 20 µl) contained 1 µl DNA template, and a final concentration of 1 × PCR buffer (containing 1.5 mM MgCl_2_), 0.4 μM of each primer, 200 μM of each dNTP and 1 U Taq DNA polymerase (MyTaq, Bioline). Thermocycling consisted of 95°C for 5 min followed by 35 cycles of 95°C for 30 s, 55°C for 30 s and 72°C for 30 s, with a final elongation step of 72°C for 7 min (Gene Amp PCR system 9700 Thermocycler, Applied Biosystems). The resulting amplicons were purified using a GenElute PCR purification kit (Sigma) and quantified with a Quant‐iT dsDNA assay kit (Thermo Fisher Scientific) with a NanoDrop 3300 fluorospectrometer (Thermo Fisher Scientific) according to the manufacturer's instructions. Target abundances were calculated using the Avogadro constant as described by Beddow et al. (2017). The DNA template concentration used in qPCR standard curves ranged from 10^2^ to 10^7^ target copies/µl and where run in triplicate, at least three no template controls (NTCs) were included on each plate. Samples were quantified in duplicate (technical replicates) with all samples from each experiment on the same plate using a Bio‐Rad CFX96 Touch Real‐Time PCR Detection System (Bio‐Rad Laboratories). Each 20‐µl reaction contained 1 µl DNA template, 1× SensiFASTTM SYBR No‐ROX dye (Bioline Reagents) and 100 nM of each primer, prepared in BrightWhite 96‐well plates (Star labs). Thermocycling consisted of 95°C for 3 min followed by 40 cycles of 95°C for 10 s and 55°C for 30 s. Copy numbers were quantified against the standard curves (*R* ^2^ > 0.99 with efficiencies of between 70% and 95%) using cfx manager software (Bio‐Rad Laboratories) with automatic settings for Cq values and the baseline.

### 2.3 DNA sequencing

PCR was carried out on each sample (including six blank extractions) using Illumina adapters and 16S rRNA V3–4 primers, forward Primer S‐D‐Bact‐0341 = 5′‐CCTACGGGNGGCWGCAG and reverse Primer S‐D‐Bact‐0785‐a‐A‐21 = 5′‐GACTACHVGGGTATCTAATCC. Thermocycling consisted of 95°C for 5 min followed by 30 cycles of 95°C for 30 s, 55°C for 30 s and 72°C for 30 s, with a final elongation step of 72°C for 7 min (Gene Amp PCR system 9700 Thermocycler, Applied Biosystems). PCR mixtures (total 25 µl) contained 5 µl DNA template, and a final concentration of 1 × PCR buffer (containing 1.5 mM MgCl_2_), 0.4 μM of each primer, 200 μM of each dNTP and 1 U Taq DNA polymerase (MyTaq, Bioline). The PCR products were cleaned using AMPure XP beads (Beckman Coulter) according to the manufacturer's instructions. Sample‐specific combinations of pairs of 8‐base indexes (Nextera XT, Ilummina) were then attached to PCR products. Thermocycling consisted of 95°C for 5 min followed by eight cycles of 95°C for 30 s, 55°C for 30 s and 72°C for 30 s, with a final elongation step of 72°C for 5 min (Gene Amp PCR system 9700 Thermocycler, Applied Biosystems). PCR mixtures (total 50 µl) contained 5 µl cleaned PCR product from the first PCR and a final concentration of 1 × PCR buffer (containing 1.5 mM MgCl_2_), 0.4 μM of each primer, 200 μM of each dNTP and 1 U Taq DNA polymerase (MyTaq, Bioline, UK). The PCR products were then cleaned using AMPure XP beads (Beckman Coulter) and quantified with a Quant‐iT dsDNA assay kit (Thermo Fisher Scientific) with a FLUO star Omega flurospectrometer plate reader (BMG Labtech). The samples were then mixed in equimolar amounts and sequenced on one lane of a HiSeq 2500 System (Illumina) at the Earlham Institute, UK (formerly TGAC UK).

### 2.4 Statistical analysis

Statistical analysis was carried out in r (R Development Core Team, 2015) and the cited associated packages. Means testing was carried out with a linear mixed model fitted in the r package “ime4” (Bates, Machler, Bolker, & Walker, 2015). 16S rRNA gene copy numbers were log_10_‐transformed to approximate a normal distribution and experimental repeats, or samples that were temporally or spatially separated were added to the model as random effects. The model was fitted with restricted maximum likelihood and degrees of freedom was estimated by the Satterthwaite approximation with the r package “imertest” (Kuznetsova, Brockhoff, & Christensen, 2017). When random effects were not required, means testing was carried out with ANOVA, or a Student's *t* test if there was only one factor with two treatments. To test differences between individual group means pairwise comparisons with Tukey's HSD (honestly significant difference) test was used (with least square mean estimations for the mixed effects models). To compare the sampling efficiencies of impingers and air filtration in the field, a Pearson's correlation coefficient was calculated between the log_10_‐transformed 16S rRNA gene copy numbers recovered by each method (normalized for the volume of air sampled) for each of the sites.

Analysis of the sequencing data was carried out using the r package Vegan (Oksanen et al., 2015) as described by Dumbrell et al. (2017). Sequence libraries were rarefied to the smallest library size as this method has good compatibility with the statistical methods and alternative methods require operational taxonomic units (OTUs) to be present in all samples (Weiss, Boyd et al., 2017; Weiss, Xu et al., 2017). Significant differences between alpha diversity metrics was evaluated using means testing with mixed effects models, as previously described. To evaluate changes in bacterial community composition (beta diversity), a distance matrix using the Jaccard index was calculated and visualized with nonmetric multidimensional scaling (NMDS). To test for differences between sampling groups, permutation‐based multivariate analysis of variance (PERMANOVA) on the distance matrix was carried out with 1,000 randomizations (Anderson & Walsh, 2013). For all tests, an alpha value of *p* < 0.05 was used.

## 3 RESULTS

### 3.1 Experiment 1: Does the filter material affect DNA yield?

**Figure 3:**
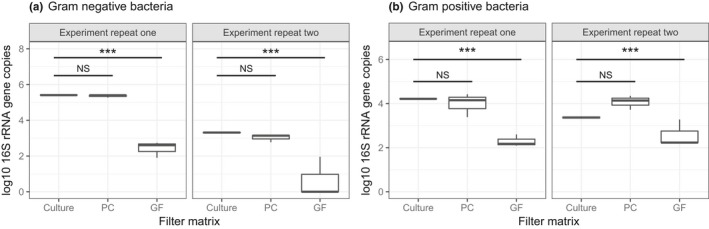
Recovery of Gram‐negative bacteria (a) or Gram ‐positive bacteria (b) from different filter matrices. GF: glass fibre filters; PC: polycarbonate filters, and Culture = direct addition of bacterial culture to a lysis tube for comparison. Each experiment was repeated, with the repeats shown on separate facets. Pairwise comparisons of least square means were perfumed using Tukey's HSD test, and the horizontal lines show significance levels between groups (NS = not significant, *p < 0.05, **p < 0.01, ***p < 0.001). The median is marked by the line that divides the boxes, the top and bottom of the box are the 75th and 25th percentiles respectively, and the whiskers shows the minimum and maximum values (*n* = *3*). Gel filters showed visible bands at the expected amplicon size (230 bp) on 1% (w/v) agarose gel after PCR and were excluded

The type of filters significantly influenced the recovery rates for Gram‐negative (*E. coli)* (Figure 3a, *F*_2,12_ = 49.7, *p* < 0.001) and Gram‐positive (*B. subtilis*) (Figure 3b, *F*_2,12_ = 22.2, *p* < 0.001) bacteria. PC filters significantly outperformed GF filters, recovering approximately three orders of magnitude more 16S rRNA gene copies for Gram‐positive and one order of magnitude for Gram‐negative bacteria (Figure 3). In addition, the recovery by PC filters was not significantly different to that of direct addition of the same amount of culture to a lysis tube for either Gram‐negative or Gram‐positives (Gram‐negatives, *t*_12_ = 0.4, *p* = 0.6; Gram‐positives, *t*_13_ = 1.6, *p* = 0.12). No background contaminants were detected in the blanks (all had Ct values within the range of the NTCs during qPCR). The exception to this was the Gel filters, which showed visible bands at the expected amplicon size (230 bp) on 1% (w/v) agarose gel after PCR and these were excluded (Supporting information Figure S3).

**Recommendation:** Use PC filters combined with a phenol/chloroform extraction procedure.

### 3.2 Experiment 2: Does the liquid impingement matrix affect DNA yield?

**Figure 4:**
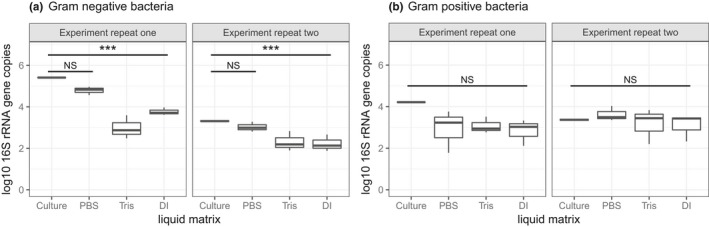
Recovery of Gram‐negative bacteria (a) or Gram‐positive bacteria (b) spiked into different liquid matrices. DI: deionized water; PBS: phosphate‐buffered saline; Tris: Tris (HCl) buffer, and Culture: direct addition of bacterial culture to a lysis tube for comparison. Each experiment was repeated, with the repeats shown on separate facets. Pairwise comparisons of least square means were perfumed using Tukey's HSD test, and the horizontal lines show significance levels between groups (NS = not significant, *p < 0.05, **p < 0.01, ***p < 0.001). The median is marked by the line that divides the boxes, the top and bottom of the box are the 75th and 25th percentiles respectively, and the whiskers show the minimum and maximum values (*n* = 3)

Liquid matrix had a significant effect on DNA recovery for Gram‐negative bacteria (Figure 4a, *F*_3,17_ = 15.4, *p* < 0.001). PBS outperformed DI and Tris (HCl) with no significant difference in 16S rRNA gene copy number between PBS and direct addition of culture (*t*_17_ = 1.0, *p* = 0.32). Both DI and Tris (HCl) recovered 1.2 and 1.5 orders of magnitude fewer 16S rRNA gene copies, respectively (DI, *t*_17_ = 4.3, *p* < 0.001; and Tris (HCl), *t*_17_ = 5.6, *p* < 0.001). In contrast, for Gram‐positive bacteria, there was no significant effect of liquid matrix for DNA recovery (Figure 4b, *F*_3,18_ = 0.9, *p* = 0.5). No background contaminants were detected in the blanks (all had Ct values within the range of the NTCs during qPCR).

**Recommendation:** Use PBS as a liquid impingement matrix as other buffers may potentially bias against Gram‐negative bacteria in mixed communities.

### 3.3 Experiment 3: What is the best way to recover bacteria from liquid impingement samples: filtering or centrifugation?

**Figure 5:**
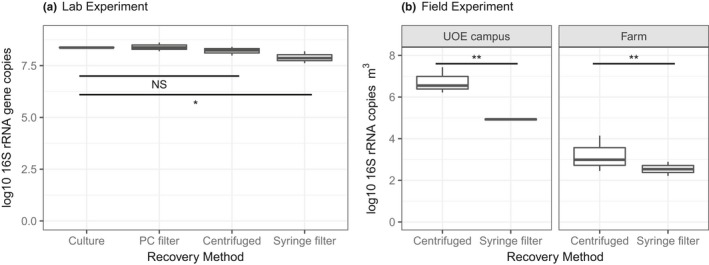
Recovery of bacteria from liquid impingement samples using different methods. The lab (a) and field (b) experiment. The facets on (b) separate the two sampling locations (University of Essex campus, and an arable farm). Pairwise comparisons of least square means were perfumed using Tukey's HSD test, and the horizontal lines show significance levels between groups (NS = not significant, *p < 0.05; **p < 0.01; ***p < 0.001). The median is marked by the line that divides the boxes, the top and bottom of the box are the 75th and 25th percentiles respectively, and the whiskers show the minimum and maximum values (*n* = 3)

**Figure 6:**
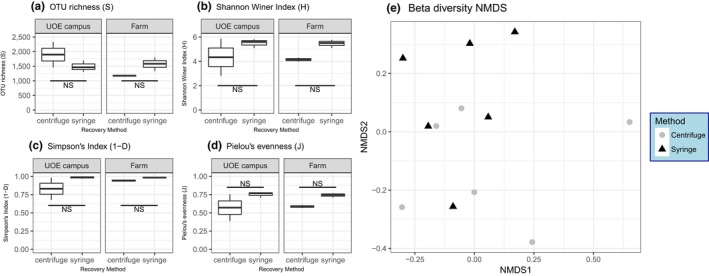
Effect of recovery method from impingement samples on bacterial alpha diversity measures (box plots, a–d) and community composition (NMDS, e) from air samples collected by liquid impingement. In (a) to (d) (alpha diversity) the sampling sites are separated by facets. Pairwise comparisons of least square means were perfumed using Tukey's HSD test, and the horizontal lines show significance levels between groups (NS: not significant, *p < 0.05; **p < 0.01; ***p < 0.001). The median is marked by the line that divides the boxes, the top and bottom of the box are the 75th and 25th percentiles respectively, and the whiskers show the minimum and maximum value (*n* = *3*). For (e) (NMDS) grey circles indicate recovery by centrifugation and black squares recovery by syringe filters [Colour figure can be viewed at http://wileyonlinelibrary.com]

In the lab experiment, the syringe filters recovered significantly fewer 16S rRNA gene copies compared to the other methods (PC filters or centrifugation to recover a bioaerosol pellet; Figure 5a, *t*_3_ = 2.8, *p* = 0.02). In the field experiment, syringe filters recovered significantly less than centrifugation by 1.2 orders of magnitude (Figure 5b, *F*_1,9_ = 11.6, *p* = 0.008). Despite the differences in DNA recovery between the two methods (syringe filters vs. centrifugation), there was no significant difference in microbial alpha (OTU richness [Figure 6a] *F*_1,8_ = 0.03, *p* = 0.85, Shannon Wiener Index [Figure 6b] *F*_1,8_ = 1.5, *p* = 0.24, Simpsons Index [Figure 6c] *F*_1,8_ = 1.83, *p* = 0.21, and Pielou's evenness [Figure 6d] *F*_1,8_ = 2.01, *p* = 0.19) or beta diversity (Figure 6e, PERMANOVA, *F*_1,8_ = 1, *p* = 0.5, *R* ^2^ = 0.1). However, the relative abundance of Enterobacteriales was twofold higher in the centrifugation treatment than the syringe filter treatment, so there may have been subtle differences in community structure driven by some taxa (Supporting information Figure S2). No background contaminants were detected in the blanks (all had Ct values within the range of the NTCs during qPCR and none of the sequences recovered from blanks was of high enough quality to form contiguous reads (see Supporting information Appendix S1).

**Recommendation:** Use centrifugation to recover bacteria from liquid impingement samples. However, syringe filters can be considered for use in the field.

### 3.4 Experiment 4: How long and what volume of air should be sampled to obtain sufficient DNA yields for downstream molecular processing in different environmental contexts?

**Figure 7:**
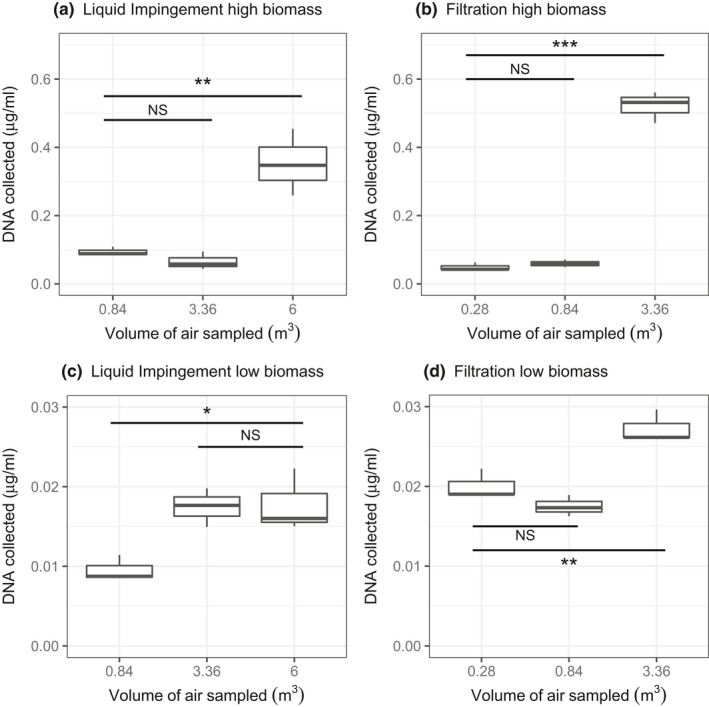
Comparison of DNA yields for varying volumes of air collected with air filtration and liquid impingement. (a, b) Environment with high biomass; (c, d) an environment with low biomass. Pairwise comparisons of least square means were perfumed using Tukey's HSD test, and the horizontal lines show significance levels between groups (NS = not significant, *p < 0.05; **p < 0.01; ***p < 0.001). The median is marked by the line that divides the boxes, the top and bottom of the box are the 75th and 25th percentiles respectively, and the whiskers show the minimum and maximum value (*n* = 3)

For all conditions, there was a significant increase in DNA recovery with increased time/volume of air sampled (Figure 7): filters at high bioaerosol concentration (*F*_2,6_ = 276.5, *p* < 0.001) and filters at low bioaerosol concentration (*F*_2,6_ = 24.8, *p* = 0.001), for impingers at high bioaerosol concentration (*F*_2,6,_ = 21.8, *p* = 0.002) and for impingers at low bioaerosol concentration (*F*_2,6_ = 8.2, *p* = 0.02). Current guidelines for preparation of libraries for PCR‐free metagenome sequencing with the Nextera DNA Library Prep Reference Guide (Illumina) are 50 ng of DNA per sample. Our best cases recovered less than this threshold, for example 13 ng (*SD* 1.1) dsDNA when sampling with filters for 120 min, and 9 ng (*SD* 2) dsDNA with impingers for 20 min respectively from a high‐biomass environment. The yields achieved here would be sufficient for metagenomics using the Nextera XT kits (Illumina), which requires 1 ng DNA per sample (according to current guidelines) or metabarcoding with a dual PCR approach using the Nextera XT indices (Illumina). However, pooling of samples would be required for an amplification‐free metagenome sequencing strategy.

**Recommendation:** Sample for at least 120 min (3.36 m^3^ air) with filters, and 20 min (6 m^3^ air) with impingement for metagenomics or metabarcoding approaches, but pooling of samples may still be required for an amplification‐free metagenome sequencing strategy.

### 3.5 Experiment 5: How do DNA yields vary between air filtration and liquid impingement sampling methods processing in different environmental contexts?

**Figure 8:**
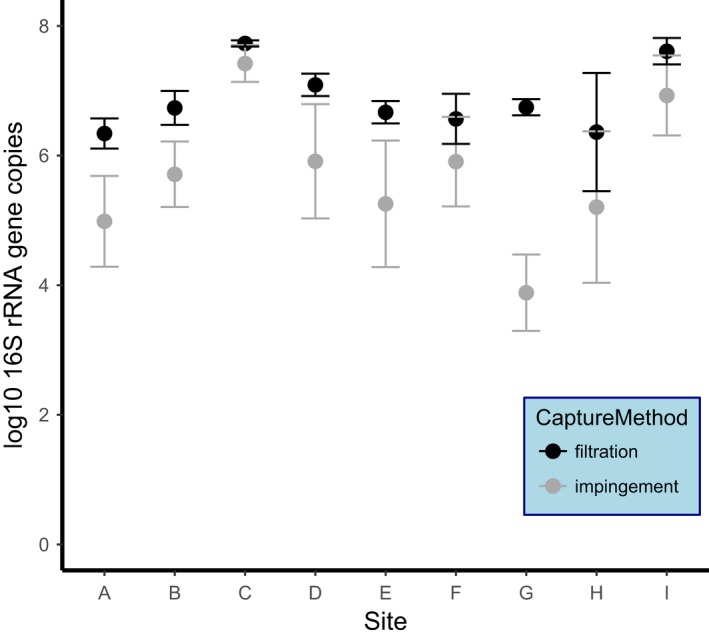
Comparison of 16S rRNA gene copies recovered per m air with filters (black) and liquid impingement (grey) from a range of sites with varying environmental conditions (see Supporting information Table S1). The mean value is marked by the point and the whiskers are the minimum and maximum range for each site (*n* = 9) [Colour figure can be viewed at http://wileyonlinelibrary.com]

Filters recovered significantly more 16S rRNA gene copies than liquid impingement by just over an order of magnitude (Figure 8, *F*_1,8_ = 23.9, *p* = 0.001). However, the difference was systematic as results for both methods were significantly correlated (Spearman correlation = 0.78, linear fit = *t*_7_ = 3.3, *p* = 0.01). The exception to this was site G, which showed a difference of over three orders of magnitude in mean 16S rRNA gene copies between sampling methods. Indeed, the *R* ^2^ of the fitted model increases from 0.55 to 0.9 if site G is excluded from the analysis.

**Recommendation:** Use air filtration for the highest recovery, but use snapshot sampling with impingement when fine‐scale temporal/spatial data are needed.

## 4 DISCUSSION

**Figure 9:**
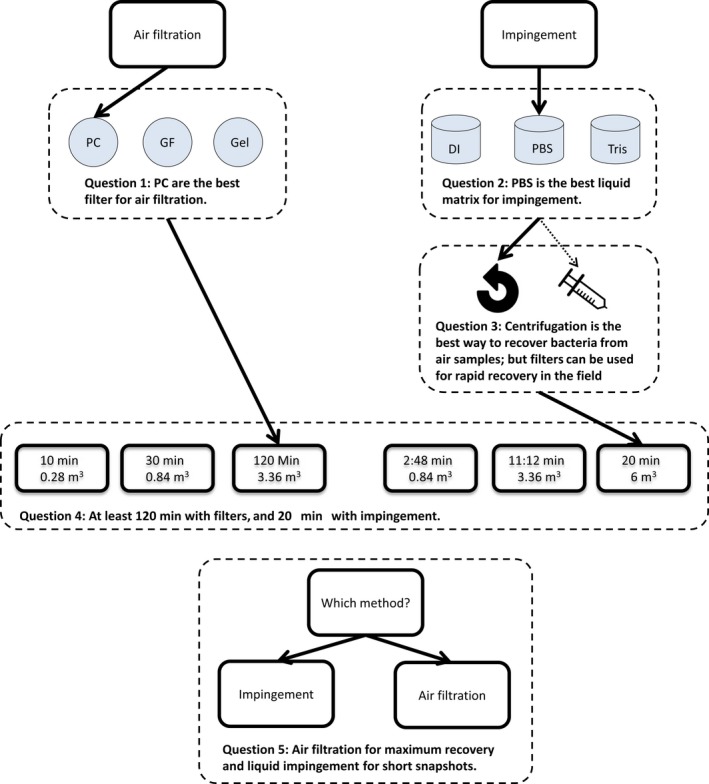
Decision flow chart for selecting the optimal air sampling method based on the results in this study [Colour figure can be viewed at http://wileyonlinelibrary.com]

In this study, we evaluated different sampling methods for collecting airborne bacteria from a range of different environments with the aim of maximizing DNA yield. Based on our results, we have made a number of recommendations for selecting the most appropriate bioaerosol sampling method (summarized in Table 2 and Figure 9).

**Table 2:** Summary of best practice, according our results, and the pros and cons of different sampling methods

Method	Best matrix	Pros	Cons	Application
Filtering	PC filter	Higher numbers collected	Long sampling time	Accuracy and total coverage
				Average background community
Impingement	Phosphate‐buffered saline	Short sampling time	Lower numbers	Short snapshots
			Evaporation prevents long sampling times	Temporal/spatial resolution
				Detect rare community members

Under the conditions tested, it is possible to collect sufficient bacterial genetic material from bioaerosols for molecular analysis across different environments (Experiments 4 and 5). Of the matrices tested, PC filters and PBS had the highest DNA recovery rates for air filtration and liquid impingement respectively. Centrifugation was also a better method for recovering bacterial bioaerosols from impingement liquids than syringe filters in terms of DNA yield. However, as diversity is not significantly affected, syringe filters are a good option in certain circumstances (e.g., for rapid in‐field analysis). Furthermore, we recommend air samples should be collected for at least 120 min (3.36 m^3^ air) with filters and 20 min (6 m^3^ air) with impingement. However, pooling of multiple samples may still be required for amplification‐free metagenomics.

### 4.1 Choice of filter for air sampling

Our results show that the type of filter used is a key consideration, as recovery rates differed by more than an order of magnitude between filter types, probably due to differential release of bacteria from the filter during DNA extraction. Other studies also concluded that recovery of bioaerosols from the sampling matrix can be a major limitation for both bacteria and fungi (Adams, Tian et al., 2015; Aguayo et al., 2018; Castaño et al., 2017; Wang et al., 2015). On flat filters (e.g., PC) the particles are easy to recover as they remain on the filter surface. In contrast, particles are not easily released from fibrous filters (e.g., GF) where they are trapped between filaments. In addition, during extraction, PC filters are dissolved in phenol/chloroform, releasing the bacteria and ensuring efficient bead lysis. GF filters, however, are more robust and do not disintegrate in phenol/chloroform, consequently obstructing bead lysis. Some studies have mitigated the lower recovery rates from GF filters by cutting them up (Cao et al., 2014; Pankhurst et al., 2012) or vortexing the filters to resuspend the bacteria into a liquid prior to DNA extraction (Be et al., 2014; Madsen, Zervas, Tendal, & Nielsen, 2015). However, to date, there is no information available on the efficiency of vortexing for cell recovery from filters. It also seems an unnecessary extra step, unless there is a specific reason not to use a flat face filter. Although membrane filters entrap particles within the filter, unlike fibrous filters they have shown good performance for bioaerosol collection elsewhere (Clark Burton, Adhikari, Grinshpun, Hornung, & Reponen, 2005; Duquenne et al., 2018; Dybwad et al., 2014a; Li, 2011), occasionally outperforming flat face filters (e.g., PC), such as when collecting *B. subtilis* endospores (Burton et al., 2005) or endotoxins (Duquenne et al., 2018). However, note that Burton et al. (2005) measured the loading capacity of the filter, rather than the ability to release particles. As bioaerosol concentrations are typically low in the environment, loading is not as important a factor as particle release. In our study, it is the combination of sampling procedure and extraction method that is crucial.

Gel filters have been used in a number of studies to collect bacterial, fungal and archaeal bioaerosols for culture‐independent analysis (Blais Lecours, Veillette, Marsolais, & Duchaine, 2012; Nehmé et al., 2009; Nehme, Létourneau, Forster, Veillette, & Duchaine, 2008; Yamamoto, Kimura, Matsuki, & Yanagisawa, 2010). We found the Gel filters were brittle and lost consistency in damp conditions, which may make them unsuitable in some environments, but enables them to release collected particles effectively. Moreover, Gel filters used in this study had trace amounts of contaminating bacterial DNA (Supporting information Figure S3), and therefore were inappropriate for culture‐independent analysis, supporting previous findings (Fahlgren, Hagstrom, Nilsson, & Zweifel, 2010). It is unclear if this extends to other filters of biological origin that are used for bioaerosol studies, such as cellulose (Adams, Bhangar et al., 2015; Bowers, McLetchie, Knight, & Fierer, 2011; Cho & Hwang, 2011). Although Gel filters may remain useful for culture or microscopy studies, we would not recommend using them for molecular methods. Our results highlight the importance of running blank extractions and field blanks (Nehmé et al., 2009) as contamination can be introduced both at the sampling stage (e.g., Gel filter contamination) or in the lab during sequence library preparation (e.g., kit contamination; Adams, Miletto, Taylor, & Bruns, 2013; Nguyen, Smith, Peay, & Kennedy, 2015).

### 4.2 Gram‐negative bias in impingement samples

For culture‐based analyses, the matrix used for impingement effects recovery of bacterial cells due to differential growth and lysis during storage at >4°C (Chang & Wang, 2015). For molecular analysis, samples are frozen, so growth is not expected to be an issue, but the differing resistance to lysis between groups of bacteria is a key consideration in any microbial ecology study (Guo & Zhang, 2013; Kennedy et al., 2014). In this study, we found significantly lower recovery of Gram‐negative bacteria when not using PBS. This indicates that Gram‐negative bacteria could be under‐represented after liquid impingement, as they are less resistant to lysis during storage (e.g., freeze‐thawing) than Gram‐positive bacteria (Salton, 1953). Once cells are lysed, genetic material is harder to collect by centrifugation due to lower mass than intact cells, resulting in the lower recovery rates observed. Using a buffer such as PBS may have reduced bacterial cell lysis during storage and associated freeze‐thawing, thus improving DNA recovery. A possible alternative to PBS could be using an additive such as Tween or glycerol (Le Goff, Bru‐Adan, Bacheley, Godon, & Wéry, 2010; Le Goff et al., 2012). For culture‐based methods, an impingement mixture containing Tween 80, peptone and Antifoam Y‐30 marginally outperformed PBS for recovery of *Staphylococcus aureus* (Chang & Wang, 2015). However, it resulted in faster rates of evaporation during sampling, which is a major limitation with impingement, and the peptone acted as a substrate for bacterial growth during storage (Chang & Wang, 2015).

### 4.3 Syringe filters versus centrifugation for recovery of cells from liquid impingement samples

In the field, centrifugation is not always logistically tractable, whereas pre‐sterilized syringe filters are easily deployed. Filters may also recover small components such as DNA and spores that are harder to recover by centrifugation making the results more representative (Mbareche et al., 2017). In this study, syringe filters recovered significantly less bacteria than centrifugation (Figure 5); however, there was no significant effect on bacterial alpha or beta diversity. Indeed, there was a nonsignificant trend towards higher diversity with the syringe filters (Figure 6b–d), supporting findings elsewhere (Mbareche et al., 2017). There were also some changes in the relative abundance, but not presence/absence, of specific taxa (e.g., increase in Enterobacteriales for syringe filters, Supporting information Figure S2). Recovery with syringe filters could be used for rapid species‐specific identification of bioaerosol agents in minutes if coupled with a portable analysis method such as loop‐mediated isothermal amplification (LAMP), or film array‐based PCR (Al‐Sheikh, 2015; Lu, Mo, Zhao, Yan, & Shi, 2011; Weller et al., 2012). It would even be possible to carry out in‐field high‐throughput sequencing, with platforms such as MinION (Oxford Nanopore Technologies, Ltd; Edwards, Debbonaire, Sattler, Mur, & Hodson, 2016; Johnson, Zaikova, Goerlitz, Bai, & Tighe, 2017). The lower total recovery rates with syringe filters need to be considered and this method would not be suitable if quantification is required. Nevertheless, in terms of diversity, syringe filters may be a suitable alternative method.

### 4.4 Is air filtration or liquid impingement the best method for air sampling?

Air filtration collected approximately an order of magnitude more 16S rRNA gene copies than liquid impingement across environmental contexts with varying bioaerosol concentrations. However, our results only considered total DNA yield and further investigation is needed to determine whether differences in microbial diversity exist between methods. The collection of specific microbial targets may also be influenced by other factors such as their dispersal mechanisms or weather conditions. Thus, information on method‐dependent patterns of microbial diversity may be vital for developing taxon‐specific sampling methods (e.g., targeting a pathogen). Frankel, Timm, Hansen, and Madsen (2012) also found that filters are more effective at collecting various bioaerosols than impingement. One possible reason for this is the relative efficiency of recovering cells from liquid versus filters. Results from Experiment 3 showed a nonsignificant trend towards lower recovery from a liquid by centrifugation than direct extraction from a PC filter. Another possibility is the cut‐off size (0.5 µm with the Coriolis µ) and decreasing collection efficiency for smaller particles with impingers (Dybwad et al., 2014a). Consequently, impingement may under‐sample smaller bioaerosols.

Collecting the highest DNA yield is not the only consideration in obtaining a representative sample. Bioaerosols can show high spatial and temporal heterogeneity, which cannot be captured with long sampling periods (Dybwad, Skogan, & Blatny, 2014b; Emerson et al., 2017). In soils, it has been proposed that a large number of low‐volume samples are preferable to a few large‐volume samples to capture high heterogeneity in microbial communities (Ranjard et al., 2003)**.** The higher sampling rates achievable with (some) impingement systems (>100 L/min), compared to air filtration (2–30 L/min), make impingement suitable for collecting snapshot samples (Blais Lecours et al., 2012; Bowers et al., 2011; Le Goff et al., 2012; Madsen et al., 2015; Pankhurst et al., 2012; Robertson et al., 2013; Shin et al., 2015; Triadó‐Margarit et al., 2016). However, caution should be taken when comparing air samples of varying duration and flow rate, as collecting 300 L over 10 min may not be the same as collecting 300 L over 2 hr and could represent fundamentally different microbial communities. For example, when a large amount of material is collected, the rare members of a community might be overlooked.

### 4.5 Determining optimal sampling time with impingement and filters

An important consideration in molecular ecology studies is how long, or what volume of air to sample. Sampling time must be sufficiently long to obtain both a representative sample and enough DNA yield for molecular analysis, without exceeding the upper quantification limit of the sampler. Our results suggest that 120 min (3.36 m^3^) with a filter or 20 min (6 m^3^) with liquid impingement is the minimum time required for metagenomic or metabarcoding applications. However, none of our conditions achieved sufficient yields for amplification‐free metagenomes (50 ng DNA per sample for Nextera Illumina), which is preferable as it avoids PCR bias. Either pooling of technical replicates, or a strategy that requires less DNA input such as Nextera XT (1 ng of DNA) would be required. It is unclear if increasing the flow rate or sampling period would be an appropriate strategy to increase DNA yield. We found that doubling the flow rate to 600 L/min with impingers did not significantly increase DNA yields (Supporting information Table S2). Either there is a trade‐off with efficiency at higher flow rates, or 300 L/min was already sufficient to collect the available material. In addition, it may not be appropriate to increase the sampling time with filters due to sample desiccation. For example, Luhung et al. (2015) found that with long periods the bacteria on filters degraded quickly, and as a result the DNA yield did not increase with time and the sample was only representative of the bacteria collected during the latter stages of sampling. This is especially important for determining microbial functional activity with RNA analyses, as RNA is degraded quickly and the bacteria will start to transcribe genes related to this stress.

The variable nature of bioaerosol concentrations over very short time scales (minutes) also needs to be considered. Our results in Experiment 4 show that the increase in yield is not directly proportional to the time sampled (Figure 7). Bioaerosol concentrations, rather than being constant, are liable to sudden and short peaks of high concentrations. Supporting information Figure S4 shows a theoretical representation of changes in bioaerosol concentration over time. Sudden peaks may occur during shorter sampling periods (shown by the grey dashed boxes) and can result in higher bioaerosol concentrations. Thus, the occurrence of high‐concentration events may be more important than sampling duration/volume in determining the amount of genetic material collected.

### 4.6 Assessing health risk of bioaerosols with molecular methods

The negative health effects of bioaerosols are a large driver for their research (Douwes et al., 2003). However, determining the health relevance of data from molecular bioaerosol studies is a challenge. Molecular methods are liable to false positives as they have low detection limits and collect genetic material from dead cells. Often the call from regulators is that we should only be interested in “viable” microorganisms, by which they mean culturable. It is a misconception that the subset of the community that is culturable reflects the active/infective microorganisms. Strategies that attempt to combine culture‐based and molecular methods should be treated with caution as they combine biases inherent to both (Duquenne, 2018). Rather, we could look to RNA‐based methods, such as metatranscriptomics, to determine the active proportion of the community.

A common way of assessing the health relevance of air pollution is to collect material from the inhalable/respirable size range (e.g., ISO 7708:1995; International Standards Organisation (ISO) 1995). The PC filter method recommended here could be used with an IOM Multidust sampler head to select for health‐relevant fractions (e.g., inhalable, thoracic or respirable) to comply with the M9 guidance for bioaerosol sampling in the UK (Environment Agency, 2018). For impingement samples, health ‐relevant fractions could be selected by using syringe filters of different pore size, similarly to Kesberg and Schleheck, (2013). However, this may not be a good strategy as impinger collection efficiency drops off at the particle sizes relevant to human health, namely <5 µm (Dybwad et al., 2014a). Best practice for differentiating the health‐relevant fractions in bioaerosols for molecular analysis is currently unknown and requires further research; however, the more pressing question is at what concentration of a specific agent does it become relevant for human health? Our ability to detect pathogenic bioaerosols with molecular methods is improving rapidly, but we cannot utilize these data unless we know what concentrations are meaningful from a health perspective.

### 4.7 Limitations of the study

In this study, not all available sampling methods were tested. For example, we used only one representative of each class of filter and therefore we can only make recommendations based on the conditions we tested. However, we do present methods for field sampling with filters or impingers that work across a wide range of environmental contexts (e.g., levels of biomass, different inhibitors and weather conditions). We aimed to optimize methods based on obtaining the maximum DNA yield possible, as low bioaerosol concentration across environmental settings is currently a key obstacle (Aguayo et al., 2018; Castaño et al., 2017). Determining the total concentration of bioaerosols accurately is important to public health. However, we are unable to draw any conclusions with respect to recovering maximal microbial diversity. Optimizing sampling based on obtaining the maximum bacterial diversity alone would not have been sufficient for developing the quantitative sampling methods required for public health monitoring. Notwithstanding this, the effects of sampling methods on diversity need further investigation.

We used pure cultures of bacteria as surrogate pathogens to test bioaerosol sampling procedures. *Escherichia coli* and *Bacillus subtilis* were selected as they are commonly found in bioaerosols (Degois et al., 2017; Dubuis et al., 2017; Pankhurst et al., 2012). However, it is unknown if these bacteria would behave in the same way when part of bioaerosol communities or when in combination with airborne contaminants. The structure of bacterial bioaerosol communities varies between sites/seasons, as does the concentration and composition of particulate matter. Different concentrations or types of particulate matter may repress the release of bacteria from filters and inhibit molecular analysis. One option is to use chamber studies, which are ideal for assessing collection efficiency, to mimic the behaviour of bacteria in bioaerosols (Carvalho et al., 2008; Dybwad et al., 2014a; Miaskiewicz‐Peska & Lebkowska, 2012). However, it is difficult to control bacterial concentrations for accurate quantification when using chambers, which was important for the goals of this study. Furthermore, chamber studies do not truly represent the form of environmental bioaerosols and the range of weather conditions and contaminants that may be found. Despite these limitations, we have shown (Experiments 4 and 5) that our methods can be translated to the field to recover genetic material across a range of environmental contexts.

## 5 CONCLUSIONS

Air filtration using PC filters gives the greatest DNA recovery, as air filtration collected an order of magnitude more bacteria per m^3^ of air sampled in comparison with impingement. Therefore, PC gives the “best” quantitative data, but due to the long sampling times required, this method may average out temporal variations. In contrast, given the faster sampling rates with impingement, we recommend this method for fine‐scale temporal/spatial ecological studies. With impingement, the liquid matrix should be PBS to reduce possible biases in recovering Gram‐negative and Gram‐positive bacteria. The optimal way to collect bacteria from the liquid matrix is centrifugation. However, for rapid recovery and on‐site analysis in the field, syringe filters are a viable alternative. Importantly, it is not the sampling procedure alone, but the combination of the sampling procedure and extraction method that is crucial. Although we assessed bioaerosol sampling across different environments (Experiments 3–5), further method optimization is needed to cover other environments and to consider microbial diversity and DNA yield together. Ultimately, molecular ecologists need to consider the conditions of their specific environment in conjunction with their study aims in order to make an informed decision of which methods to use and this study provides a resource to facilitate this.

## AUTHOR CONTRIBUTIONS

I.C., A.J.D., C.W. and F.C. conceived the original project. R.M.W.F. performed all the experimental work and data analysis. S.G.‐A. assisted with the field sampling. All authors contributed to writing the paper and approved the final manuscript.

## Data Availability

Sequences from this study are available through the European Nucleotide Archive under Project accession number PRJEB26329 and the individual sequences are ERS2414111‐–23. All other data generated or analysed during this study are included in the Supporting information Appendix S1 files.
