# Supplementary material for: Bioaerosol biomonitoring: Sampling optimization for molecular microbial ecology
Source: Mol Ecol Resour. 2019 Apr 20;19(3):672–90. doi: 10.1111/1755-0998.13002 (PMC6850074; doi:10.1111/1755-0998.13002)
Supplement: Supplementary file 1 [file MEN-19-672-s001.pdf]

MOLECULAR ECOLOGY RESOURCES

Supplemental Information for:

Bioaerosol Biomonitoring: Sampling Optimisation for Molecular Microbial Ecology.

Robert M.W. Ferguson[1], Sonia Garcia-Alcega[2], Frederic Coulon[2], Alex J. Dumbrell[1], Corinne Whitby[1], Ian Colbeck[1*]

[1]University of Essex, School of Biological Sciences, Wivenhoe Park, Colchester, CO4 3SQ, UK
[2]Cranfield University, School of Water, Energy and Environment, Cranfield, MK43 0AL, UK
*Corresponding Author: Prof Ian Colbeck, School of Biological Sciences, Wivenhoe Park, Colchester, CO4 3SQ, UK. Tel: +44 1206 872203; Email: colbii@essex.ac.uk

Table of Contents:

**Bacterial culture growth conditions**	Page 2
**DNA extraction**	Page 2
**Sterilisation of plastics with Milton**	Page 2-3
**Figure S1.**	Page 3
**Bioinformatics**	Page 4-5
**Figure S2.**	Page 5
**DNA contamination in gelatin filters**	Page 6
**Figure S3.**	Page 6
**Figure S4.**	Page 7
**Table S1. Summary of sampling sites used in Experiment 5**	Page 8
**Table S2. Summary of DNA yields from field sampling**	Page 9
**References**	Page 10
**Raw data for experiment 1**	Separate CSV file
**Raw data for experiment 2**	Separate CSV file
**Raw data for experiment 3**	Separate CSV file
**Raw data for experiment 4**	Separate CSV file
**Raw data for experiment 5**	Separate CSV file

MOLECULAR ECOLOGY RESOURCES

Bacterial culture growth conditions: Cultures of *Escherichia coli* DH5α or *Bacillus subtilis* (obtained from Essex Culture Collection) were grown in Luria-Bertani (LB) broth with shaking (30 rpm) at 30°C for four days. Growth was measured by daily turbidity measurements at 470 nm (Jenway 7300 spectrophotometer, Bibby Scientific, UK). Cell density was confirmed by spreading 50 µL of culture on 1.5% (w/v) LB agar plates in a serial dilution and counting distinct colonies after 3 days incubation at 30°C.

DNA extraction: DNA extraction was carried out with 1% (v/v) sodium dodecyl sulphate (SDS) buffer [10mM Tris-HCl pH 8, 25mM Na_2EDTA pH 8, 100mM NaCl] (Sigma, UK). Filters or bioaerosol pellets from impingement were placed in bead-beating tubes with 500 µL SDS buffer. The tubes were incubated at 70°C for 45 mins in a water bath and then cooled on ice. Phenol/chloroform/isoamyl alcohol (25:24:1, 500 µL) at pH 8.0 (Sigma, UK) was added to the bead-lysis tubes and bead beated for 2 x 30 s at 6800 min^{-1} (Precellys Evolution, Bertin Technologies, France). The tubes were then centrifuged at 16,000 x *g* for 5 mins. The aqueous layer was removed and nucleic acids were precipitated overnight at room temperature with 100% (v/v) isopropanol (Fisher, UK). DNA pellets were centrifuged at 16,000 x *g* for 30 mins, the pellets washed with ice-cold ethanol 70% (v/v), air-dried at room temperature for approximately 20 min, re-suspended in 25 µL of ultrapure sterile water and stored at -20°C.

Sterilisation of plastics with Milton: Plastic Coriolis sampling cones were contaminated with pure cultures of *B. subtilis* by picking up approximately 10 colonies

MOLECULAR ECOLOGY
RESOURCES

from an agar plate and spreading them on the inside of the cone with a tooth-pick. These cones were then sterilised with Milton's sterilising solution for 15 minutes. Once dried, 10 ml sterile deionised water was added to the cone and vortexed for 2 x 45 secs. The DI water was then centrifuged at 3395 x *g* for 45 mins to recover a pellet and subjected to DNA extraction as detailed before. The 16S rRNA gene was amplified from the purified DNA under the following cycling conditions, 95°C for 5 mins followed by 35 cycles of 94°C for 30 secs, 55°C for 30 secs and 72°C for 30 secs, and elongation at 72°C for 7 mins. Figure S1 shows the PCR products obtained after gel electrophoresis on 1% (w/v) 1 x TAE agarose gel stained with ethidium bromide 10 mg L^{-1}.

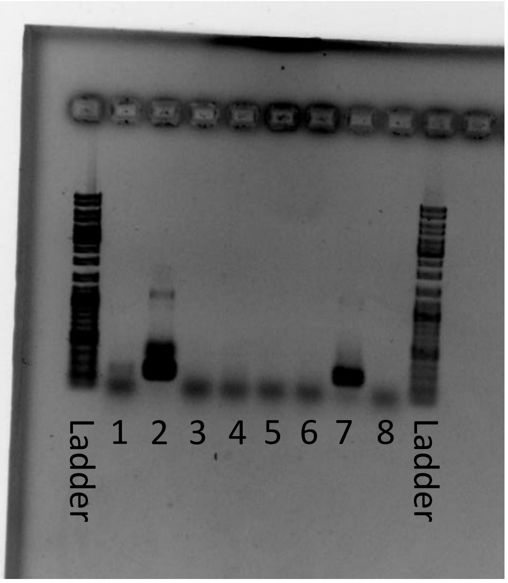

Figure S1. Lanes 1 = negative control, 2 = positive control, 3 = contaminated cone after sterilisation, 4 = contaminated cone after sterilisation, 5 = cone fresh from sterile package, 6 = cone fresh from sterile package after sterilisation, 7 = contaminated cone with no sterilization, and 8 = DI water used for DNA recovery. Ladder = GeneRuler DNA ladder Mix SM0333 (ThermoScientific, UK).

MOLECULAR ECOLOGY RESOURCES

Bioinformatics: A total of 558,331 sequence reads were obtained and processed on QIIME (Caporaso et al., 2010) and standalone packages as detailed in (Dumbrell et al. 2017). First quality filtering was carried out with Sickle (Joshi & Fass, 2011) with trimming when the average Q score dropped under 30 across a sliding window of 35 base pairs; then error correction was carried out with SPAdes (Bankevich et al., 2012) and BayesHammer (Nikolenko, Korobeynikov, & Alekseyev, 2013) . The paired sequences were then joined with PEAR (Zhang, Kobert, Flouri, & Stamatakis, 2014) implemented in PANDAseq (Masella, Bartram, Truszkowski, Brown, & Neufeld, 2012). Further quality filtering was carried out in QIIME to remove sequences with ambiguous (N) bases and homopolymer inserts longer than 8. The sequences were then clustered *de novo* into operational taxonomic units (OTUs) at the 97% level using VSEARCH (Rognes, Flouri, Nichols, Quince, & Mahé, 2016). Chimeric sequences were then removed *de novo* chimera checker UCHIME (Edgar, Haas, Clemente, Quince, & Knight, 2011). The representative sequence from each OTU were assigned a taxonomic group using the RDP classifier algorithm (Wang, Garrity, Tiedje, & Cole, 2007). One sample had < 200 reads and was excluded from further analyses, excluding this the average read depth per sample was 50,740 (SD 49405.21). Blank extractions were also sequenced in parallel; none of the sequences were of sufficient quality to make contiguous reads. In addition none of the sequences obtained from the blank extractions matched known bacterial taxa when the forward only reads were put though the bioinformatics pipeline described above. Analysis of the sequencing data was

MOLECULAR ECOLOGY
RESOURCES

carried out using the R package Vegan (Oksanen et al., 2015). Figure S2 shows a summary of the dominant bacterial taxa detected.

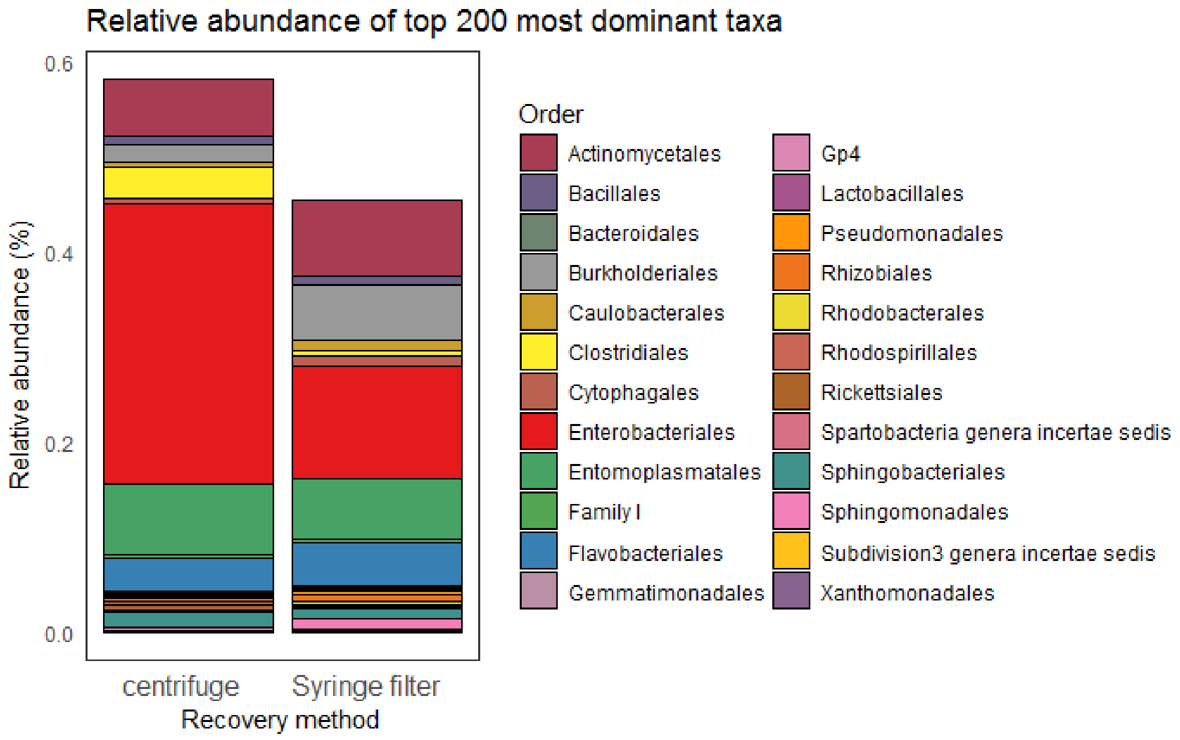

Figure S2. Relative abundance of the top 200 dominant bacterial taxa recovered by filters and centrifugation at order level, Experiment 3.

MOLECULAR ECOLOGY RESOURCES

DNA contamination in gelatine filters: To determine if there was any DNA contamination, DNA **was** extracted from Gel filters (sterile from packet) as previously described (Figure S3, lanes 4-8). For comparison the Gel fitters were also spiked with *E. coli* (Figure S3, lanes 9 and 10) and a blank extraction (Figure S3, lane 2) was also carried out. The 16S rRNA gene was amplified from the purified DNA under the following cycling conditions, 95°C for 5 mins followed by 35 cycles of 94°C for 30 secs, 55°C for 30 secs and 72°C for 30 secs, and elongation at 72°C for 7 mins. Figure S3 shows the PCR products obtained after gel electrophoresis on 1% (w/v) 1 x TAE agarose gel stained with ethidium bromide 10 mg L^{-1}. Clear bands were detected in lanes 7-8 and faint bands in lanes 5-6, indicating the Gel filters contained DNA and are therefore unsuitable for molecular applications.

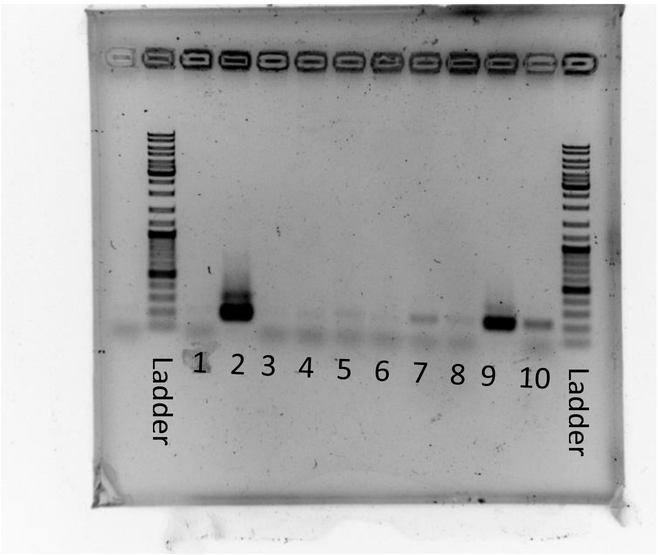

Figure S3. Lanes 1 = negative control, 2 = positive control, 3 = blank extraction, 4 = PC filter, 5 = “sterile” gelatine filter, 6 = “sterile” gelatine filter, 7 = “sterile” gelatine filter, 8 = “sterile” gelatine filter 9 = gelatine filter + *E. coli*, and 10 = gelatine filter + *E. coli*. Ladder = GeneRuler DNA ladder Mix SM0333 (ThermoScientific, UK).

MOLECULAR ECOLOGY RESOURCES

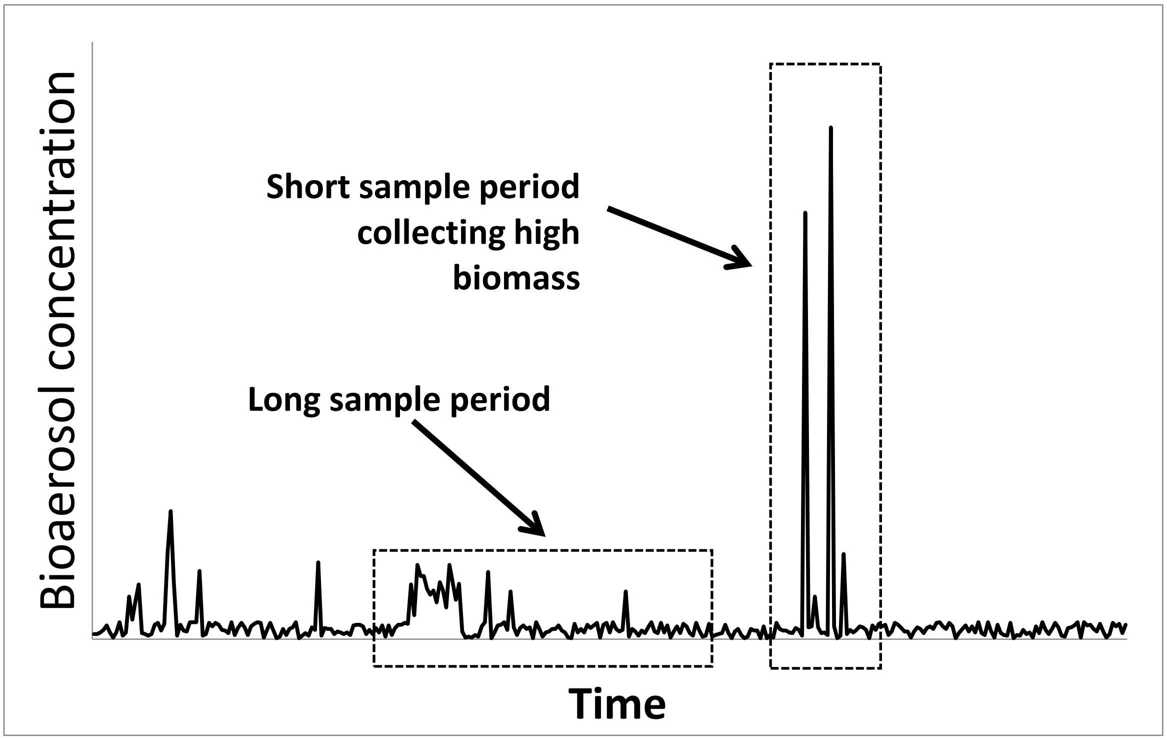

Figure S4. A theoretical representation of changes in bioaerosol concentration over time. Dashed grey boxes show differing sampling times that could lead to higher concentrations in the shorter time period.

MOLECULAR ECOLOGY RESOURCES

Table S1. Summary of sampling sites used in Experiment 5. * denotes Waste Water Treatment Facility, ** denotes Mechanical Biological Treatment Facility.

Site	Latitude	Longitude	Date (2016) m/dd	Type	Temp (°C)	Relative humidity (%)	Wind speed (m s-1)
A	51° 47' 41.4744'' N	0° 50' 24.0036'' E	8/27	Farm	20.0 ± 1.7	87.0 ± 9.4	2.8 ± 0.8
B	51° 52' 49.9584'' N	0° 54' 03.7728'' E	8/26	Urban	20.4 ± 1.3	63.3 ± 7.3	1.7 ± 1.0
C	51° 50' 33.9972'' N	0° 46' 27.2784'' E	7/28	Compost	18.4 ± 1.6	65.1 ± 5.6	1.8 ± 0.7
D	52° 03' 39.9780'' N	0° 38' 39.5628'' W	8/08	Farm	19.3 ± 0.8	47.9 ± 2.3	3.2 ± 1.2
E	52° 00' 59.7024'' N	0° 46' 06.5568'' W	8/10	Urban	17.9 ± 1.5	53.9 ± 6.2	1.7 ± 0.9
F	52° 04' 45.7176'' N	0° 37' 35.9508'' W	8/11	WWTP*	18.2 ± 2.0	71.5 ± 6.3	2.1 ± 1.0
G	51° 42' 54.4248'' N	0° 00' 05.4864'' E	8/22	Farm	22.0 ± 1.8	70.3 ± 4.9	3.0 ± 0.9
H	51° 32' 51.6552'' N	0° 01' 01.2288'' W	8/23	Urban	27.7 ± 1.9	46.9 ± 3.2	2.0 ± 0.9
I	51° 30' 33.6816'' N	0° 10' 27.1632'' E	8/24	MBT**	30.1 ± 3.1	45.2 ± 4.2	2.4 ± 1.2

MOLECULAR ECOLOGY RESOURCES

Table S2. Summary of DNA yield (total ng in 25 µl) from sampling with filters and impingers based on our recommendations (fig 9) at a rural location 21/7/18.

Method	Flow rate (L min-1)	Sample time (mins)	Total yield (ng DNA)
Filters	28	120	1.1 ±1 (n = 15)
Impinger	300	20	3.1 ± 5.6 (n = 24)
Impinger	600	15	4.5 ± 2.4 (n = 6)

References

Bankevich, A., Nurk, S., Antipov, D., Gurevich, A. A., Dvorkin, M., Kulikov, A. S., … Pevzner, P. A. (2012). SPAdes: a new genome assembly algorithm and its applications to single-cell sequencing. *Journal of Computational Biology : A Journal of Computational Molecular Cell Biology*, *19*(5), 455–477. http://doi.org/10.1089/cmb.2012.0021

Caporaso, J. G., Kuczynski, J., Stombaugh, J., Bittinger, K., Bushman, F. D., Costello, E. K., … Knight, R. (2010). QIIME allows analysis of high-throughput community sequencing data. *Nature Methods*, *7*(5), 335–336. http://doi.org/10.1038/nmeth.f.303

Dumbrell, A. J., Ferguson, R. M. W., & Clark, D. R. (2017). Microbial Community Analysis by Single-Amplicon High-Throughput Next Generation Sequencing: Data Analysis -- From Raw Output to Ecology. In T. J. McGenity, K. N. Timmis, & N. Balbina (Eds.), *Hydrocarbon and Lipid Microbiology Protocols: Microbial Quantitation, Community Profiling and Array Approaches* (pp. 155–206). Berlin, Heidelberg: Springer Berlin Heidelberg. http://doi.org/10.1007/8623_2016_228

Edgar, R. C., Haas, B. J., Clemente, J. C., Quince, C., & Knight, R. (2011). UCHIME improves sensitivity and speed of chimera detection. *Bioinformatics*, *27*(16), 2194–2200. http://doi.org/10.1093/bioinformatics/btr381

Joshi, N., & Fass, J. (2011). Sickle: A sliding-window, adaptive, quality-based trimming tool for FastQ files (Version 1.33).

Masella, A. P., Bartram, A. K., Truszkowski, J. M., Brown, D. G., & Neufeld, J. D. (2012). PANDAseq: paired-end assembler for illumina sequences. *BMC Bioinformatics*, *13*(1), 31. http://doi.org/10.1186/1471-2105-13-31

Nikolenko, S. I., Korobeynikov, A. I., & Alekseyev, M. A. (2013). BayesHammer: Bayesian clustering for error correction in single-cell sequencing. *BMC Genomics*, *14*(Suppl 1), S7. http://doi.org/10.1186/1471-2164-14-S1-S7

Oksanen, J., Blanchet, F. G., Kindt, R., Legendre, P., Minchin, P. R., Hara, R. B. O., … Wagner, H. (2015). Vegan: Community Ecology Package. R package version 2.3-2. https://CRAN.R-project.org/package=vegan. http://doi.org/10.4135/9781412971874.n145

Rognes, T., Flouri, T., Nichols, B., Quince, C., & Mahé, F. (2016). VSEARCH: a versatile open source tool for metagenomics. *PeerJ*, *4*, e2584. http://doi.org/10.7717/peerj.2584

Wang, Q., Garrity, G. M., Tiedje, J. M., & Cole, J. R. (2007). Naive Bayesian classifier for rapid assignment of rRNA sequences into the new bacterial taxonomy. *Applied and Environmental Microbiology*, *73*(16), 5261–7. http://doi.org/10.1128/AEM.00062-07

Zhang, J., Kobert, K., Flouri, T., & Stamatakis, A. (2014). PEAR: a fast and accurate Illumina Paired-End reAd mergeR. *Bioinformatics (Oxford, England)*, *30*(5), 614–20. http://doi.org/10.1093/bioinformatics/btt593
